# Fructose Consumption among Students at The University of Guanajuato

**DOI:** 10.5195/cajgh.2019.375

**Published:** 2019-05-10

**Authors:** Rosa Dejanira Medina Terán, Joel Ramírez Emiliano, Hilda Lissette López Lemus

**Affiliations:** 1 Department of Nursing and Obstetrics, Celaya-Salvatierra Campus, University of Guanajuato, Mexico; 2 Department of Medical Sciences, León Campus, University of Guanajuato, Mexico

**Keywords:** Fructose, Obesity, Students, University of Guanajuato

## Abstract

**Introduction:**

Fructose is a monosaccharide commonly found in fruits. However, it can also be found in carbonated beverages, cereals, fruit juices, and in other processed fruit. The consumption of fructose in moderate to high amounts increases levels of triglycerides in plasma and alters hepatic glucose homeostasis. Little information is avialble on fructose consumption in Mexico. The aim of this study was to determine the amount of fructose consumption among college students enrolled at the Unviersity of Guanajuato in Mexico (Celaya-Salvatierra Campus).

**Methods:**

This was an explorative, cross-sectional descriptive study. A total of 57 full time students attending Physical Therapy and Rehabilitation program were included in the analysis. Demographic data, food frequency questionnaire, and body mass index (BMI) were collected from all students. Data were analyzed by descriptive statistics; discrete variables were reported as frequencies or percentages and continuous variables were reported as means and standard deviations.

**Results:**

The average age of participants was 19.5 ± 2.8 years. 72% of participants were female and 28% were male. The average BMI was 24.0 ± 4.1 Kg / m^2^, indicating normal BMI range. Fructose consumption was roughly 55g per day.

**Conclusions:**

Previous research demonstrated that levels below 50 mg per day are safe. Madero et al. reported that that consumption of 50-70 g of fructose per day is considered to be moderate. We found that students consumed 55g of fructose, so their fructose intake is at a moderate level and should not be an obesity-inducing factor, also consistent with BMI ranges of our research participants. Considering obesity epidemic in Mexico, further studies examining the source of calories in Mexican poipualation are warranted, especially among young people.

